# General Belief in a Just World Is Positively Associated with Dishonest Behavior

**DOI:** 10.3389/fpsyg.2017.01770

**Published:** 2017-10-10

**Authors:** Kristin Wenzel, Simon Schindler, Marc-André Reinhard

**Affiliations:** Department of Psychology, University of Kassel, Kassel, Germany

**Keywords:** dishonest behavior, cheating, coin toss, belief in a just world, general belief in a just world

## Abstract

According to the just-world theory, people need to – or rather want to – believe that they live in a just world where they will receive what they earn and consequently earn what they receive. In the present work, we examined the influence of people’s general and personal beliefs in a just world (BJW) on their (dis)honest behavior. Given that general BJW was found to be linked to antisocial tendencies, we expected stronger general BJW to be linked to more dishonesty. Given that personal BJW was found to be correlated with trust and justice striving, a negative link with dishonesty could be assumed. In one study (*N* = 501), we applied a common coin-toss paradigm to assess dishonesty. General BJW significantly predicted the probability of tossing the target outcome, that is, higher general BJW was linked to more dishonest behavior. This effect was found to be independent from personal BJW and self-reported importance of religion. Unexpectedly, there was no significant relationship between personal BJW and levels of dishonesty. These findings imply that although BJW normally serves an adaptive function, at least the facet general BJW has maladaptive side-effects.

## Introduction

In our daily lives, we are continuously confronted with injustices – witnessed or experienced– that concern ourselves or others and simultaneously shape our own behavior. Thus, it is important to understand how people perceive injustices, how they react to them, how they give meaning to them, and also how they try to cope with them. The just-world theory (e.g., Lerner, 1977, 1980) emphasizes the adaptive function of the beliefs in a just world (BJW) to cope with injustices and inequalities. Usually, this contains aspects of believing that the world is a generally just place or that it is at least just for oneself or for others. Such coping or meaning-making mechanisms (among others) include cognitive re-evaluations of unjust events and justice-repairing through own just reactions, as well as negative and anti-social tendencies, norm-breaking behavior, and victim-blaming (e.g., Dalbert, 2009; Hafer and Sutton, 2016); the latter is especially relevant regarding persons with a strong general BJW. Regardless of the great amount of research concerning this belief and its possible negative side-effects or consequences, to our knowledge there are no empirical studies exploring the relationship between people’s belief that the world is a just place and their actual own dishonest behavior. All in all, BJW serves an adaptive function and consists of different facets (i.e., personal and general BJW). We would like to explore a possible maladaptive side-effect of general BJW: norm-breaking behavior. We try to more thoroughly explore negative aspects or consequences of a strong BJW, whereby we focus primarily on general BJW. A part of such negative aspects is norm-breaking behavior, such as dishonesty or cheating.

An interesting consideration could be that the belief in a generally just world may lead (as a maladaptive side-effect) to more dishonesty. For instance, people could use their belief in a generally just world as justification for dishonesty, because in such a world other people should get what they deserve no matter how someone else behaves; therefore, people could cheat more often, because they should not feel guilty for their dishonesty, and they would not have to suffer the moral costs of such behavior. On the other hand, a negative correlation between personal BJW and dishonesty could be assumed.

### Belief in a Just World

Lerner (1977, 1980) proposed that individuals need to believe in a just world to deal with witnessed or experienced injustice, helplessness, and insecurity. The just-world theory (Lerner, 1980) assumes that people want to believe that they live in a world where good things happen to good people and bad things only to bad ones and where therefore everyone harvests what they sow (see also Furnham, 2003; Dalbert, 2009; Hafer and Sutton, 2016). This belief is essential for people to feel safe and positive and to perceive the world as a predictable and manageable place (Lerner, 1980; Dalbert, 2009; Hafer and Sutton, 2016). Therefore, the BJW normally serves an adaptive function.

The belief in a just world is often seen as a personality trait with dispositional variations (e.g., Furnham, 2003; Dalbert, 2009; Hafer and Sutton, 2016). Literature often differentiates between varying facets of BJW and different means of measurement. This mostly includes a general BJW, a personal BJW (Dalbert, 2009), and a BJW for others (Lipkus et al., 1996). General BJW refers to the belief that the world is a just place in general, where all people normally get what they deserve, are treated fairly, and will be compensated for experienced injustices (Dalbert, 2009; Hafer and Sutton, 2016). On the contrary, personal BJW refers to the belief that oneself will be treated fairly and that one’s own life is just (Dalbert, 2009; Hafer and Sutton, 2016), which people normally endorse more strongly than the general belief (Dalbert, 1999; Hafer and Sutton, 2016). Furthermore, personal BJW can in particular be seen as an expression of social desirability (Alves and Correia, 2010) so that people deliberately give high ratings of their levels of personal BJW to distinguish themselves from others. Beyond that, they believe that stronger personal BJW conveys specific images of being likable, competent, and successful – higher levels of general BJW only led to such positive images when it was not controlled for personal BJW (Alves and Correia, 2010). A high personal BJW therefore can be viewed as a socially and normatively desirable or expected trait which is linked to normative and desirable behavior, whereas general BJW probably is not (especially considering that it could seem to be inappropriate to state that the world is just when so many injustices happen all the time). Because the general belief includes not only other people but also the self, general BJW and personal BJW seem to be two correlated but still different facets of one belief in a just world. All in all, general BJW is often related to harsh responses to other people (e.g., disadvantaged individuals or groups) and derogation of victims, which can be seen as a negative or maladaptive side-effect of BJW, whereas, in contrast personal BJW is rather linked to subjective well-being or interpersonal trust, which seems to be a positive or adaptive consequence of a high BJW (e.g., Lipkus et al., 1996; Bègue and Bastounis, 2003; for a recent overview see Hafer and Sutton, 2016). Therefore, it is important to assess both facets of BJW and to assume different effects on people‘s behavior. If general BJW is measured and personal BJW is controlled for, this should leave a BJW solely concerning other people. In line with this, the scale BJW for others refers to the belief that other people (excluding oneself) get what they deserve (Lipkus et al., 1996). General BJW and BJW for others can be viewed as strongly related: both access the belief that the world is a just place in general, the only difference being that BJW for others explicitly excludes oneself from this equation, which general BJW does not. Still, both can be seen as measuring a belief in a generally just world, so it can be assumed that prior findings concerning BJW for others should apply for general BJW as well, and findings concerning general BJW should apply for BJW for others. Although we report studies using both versions of measurements (general BJW and BJW for others), our current work focuses mostly on general and personal BJW.

General BJW often correlates with religiousness – especially with the faith in later, ultimate justice (Hafer and Sutton, 2016). General BJW and BJW for others also positively correlate with harsh social attitudes and antisocial tendencies (Bègue and Muller, 2006; Sutton and Winnard, 2007; Dalbert, 2009; Hafer and Sutton, 2016), as well as with less social activism (Hafer and Sutton, 2016). In one study (Sutton and Winnard, 2007) BJW for others even positively predicted self-reported intentions to engage in delinquent behavior (i.e., theft) and may generally encourage violations of norms (e.g., Bègue and Muller, 2006; Sutton and Winnard, 2007). For instance, Sutton and Winnard (2007) assume that people who strongly believe that the world is a just place for others may have a greater willingness to exploit and victimize others, because this belief may facilitate the perception that other people are blameworthy or unsympathetic because only such people could be victims of antisocial behavior in the first place. Due to that, people would probably not feel guilty for potential misdeeds. In line with these assumptions, Dalbert (1999) demonstrated that participants with a high personal BJW, who were aware of their own unfairness, suffered a decrease in their self-esteem because their unfair behavior contradicted the contract between them and the world to behave well– and to be treated well in return. In contrast, participants with a high general BJW who thought about their own unfairness did not suffer such a decrease. They probably do not perceive their own unfairness as consequential, as severe, or as contract-breaking. Thus, people with higher personal BJW value the aforementioned contract more than people with stronger levels of general BJW. This could lead to the assumption that people with a strong personal BJW try to behave well so that good things will happen to them, whereas people with higher levels of general BJW do not have to act this way because they do not value the contract. All in all, individuals with a high personal BJW are more sensitive toward injustices (e.g., Dalbert, 1999), strive for justice themselves (e.g., Lerner, 1977; Dalbert, 2009; Schindler and Reinhard, 2015), prefer equal allocations in dictator games (Dalbert and Umlauft, 2009), and are more committed to just means (e.g., Sutton and Winnard, 2007). Further, personal BJW is linked to interpersonal trust (Zuckerman and Gerbasi, 1977; Bègue, 2002) and to prosocial behavior (Bègue, 2014).

It may even be possible to connect theories concerning the BJW and meaning-making literature which explores the adjustment to stressful, unjust, or traumatizing events (for an overview see Park, 2010). Meaning making is often seen as a possibility to re-establish someone’s BJW after traumatic events or injustices (e.g., illnesses) which violate people’s BJW (Park et al., 2008). This serves an adaptive function because when meaning to such an event is made, for example subjective well-being, adjustment, and health increase (e.g., Park et al., 2008; Park, 2013). Meaning making also negatively correlates with aggressive, antisocial and irresponsible behavior (past and intended; Brassai et al., 2012) as well as bullying perpetration (O’Donnell, 2015). In contrast, if the meaning making is not successful, such a search for meaning can lead to negative consequences like low positive affect, an overall poorer quality of life, and more negative affect (e.g., Kernan and Lepore, 2009). That is to say there is a maladaptive side-effect. All in all, BJW and meaning making are strongly interwoven: both normally serve adaptive functions but both can also present negative or maladaptive side-effects.

Although there has been a great deal of research concerning the just-world theory, there is considerably little empirical research regarding the relationship between participants’ general or personal BJW and dishonesty as a possible (maladaptive) side-effect. We are only aware of one study (Schindler and Reinhard, 2015) which revealed that participants with a higher personal BJW performed worse in deception detection than participants with a lower personal BJW (presumably due to low motivation to detect deception).

### Dishonest Behavior

Although honesty commonly constitutes an important norm and people generally value honesty, trustworthiness, and credibility (i.e., Geißler et al., 2013), dishonest behavior can be observed throughout our daily lives (e.g., DePaulo et al., 1996). However, empirical evidence has shown that even when people have the opportunity to cheat without getting caught (and could therefore gain financially), they still try to maintain a positive self-concept and hence do not cheat to the fullest possible magnitude (e.g., Mazar et al., 2008; Shalvi et al., 2011; Fischbacher and Föllmi-Heusi, 2013; Abeler et al., 2014). As a result, people attempt to find a balance between those different motivations and often cheat a little bit (to increase their gain), but not as much as they could (so that they still sustain their positive self-concepts and avoid suffering moral costs). Thus, dishonesty is mostly shown when the gain of it outweighs the possible costs of, for instance, getting caught or feeling guilty and immoral. Dishonest behavior has been found to be related to personality traits such as honesty–humility (Hilbig and Zettler, 2015) and situational cues like activation of the norm of honesty (Mazar et al., 2008) or loss framing (i.e., Schindler and Pfattheicher, 2017). Further, dishonesty increases with justifications (e.g., Mazar et al., 2008; Shalvi et al., 2011). Such self-serving justifications (which can emerge before or after intentional (un)ethical behavior) lessen the anticipated (or the experienced) threat to the moral self through manners that balance or even reduce the costs of dishonest behavior (Shalvi et al., 2015). Therefore, people could behave dishonestly while still feeling moral and good. The authors describe different forms of justifications which reduce ethical dissonance. This even applies to people who actually highly value their morality.

### The Present Research

The present research was conducted to explore the connection between participants’ BJW and their own dishonest behavior. Individuals with a high general BJW should believe that the world is a just place in general, where every human (including others and themselves as well) receives what they earn and earn what they receive (cf. Lerner, 1980). As mentioned before, general BJW is often correlated with harsh attitudes, antisocial tendencies, and delinquent intentions (e.g., Bègue and Muller, 2006; Sutton and Winnard, 2007; Dalbert, 2009; Hafer and Sutton, 2016). BJW for others and general BJW have repeatedly been shown to enhance self-reported antisocial and norm-breaking behaviors or attitudes (Sutton and Winnard, 2007; Hafer and Sutton, 2016), which could be transferred to dishonest behavior. The aforementioned study from Dalbert (1999) supports this assumption: participants with high general BJW did not suffer a decrease in self-esteem although they had been reminded of their own unfairness, so that they presumably did not feel guilty about their own unfair behavior. Therefore, we expect higher levels of general BJW to be positively linked to dishonest behavior as a maladaptive side-effect. Besides, the belief that the world is generally just can serve as a justification for cheating (and justifications increase the amount of dishonesty because with justifications, cheating people do not suffer moral costs; e.g., Shalvi et al., 2011), since other people get what they deserve anyway, independent of someone-else’s dishonesty. This means that people with stronger levels of BJW could believe that others will get what they deserve, whether or not they themselves cheat, because their behavior has no effect on these others. Therefore, people with a strong general BJW would probably not feel guilty for dishonest behavior because their actions would not really harm other people. Thus, the moral costs of dishonesty seem to be lower for people with higher (in contrast to lower) levels of general BJW than for people with a strong personal BJW. Such lower moral costs can in turn lead to more dishonesty because then the gain would outweigh the costs. All in all, in a generally just world, people’s own dishonest behavior shouldn’t affect other people’s fates, because their lives should be just anyway, or they would be compensated elsewhere for afflicted injustices. Therefore, people may try to justify for themselves that they do not harm others with their dishonest behavior.

Taking the aforementioned findings concerning antisocial tendencies, maladaptive side-effects, and the (self-) justifications into account, we suppose that a stronger general BJW is positively connected to people’s own dishonesty. Regarding personal BJW, previous research suggests that people with a strong personal BJW are more sensitive to justice, that they strive for justice more often themselves, and that they behave more prosocially (Lerner, 1977; Sutton and Winnard, 2007; Dalbert, 2009; Dalbert and Umlauft, 2009; Schindler and Reinhard, 2015; Bègue, 2014). Thus, assuming honesty to be a matter of justice, a negative link toward dishonesty seems likely. To investigate our ideas, we assessed dispositional general and personal BJW and applied a common coin-toss paradigm to assess participants’ actual dishonest behavior. Additionally, we controlled for self-reported importance of religion.

## Materials and Methods

### Participants

Coin-toss paradigms often reduce power, because they can add random noise to the response (Ulrich et al., 2012). Therefore, compared to different analyses, larger samples are required to obtain the same level of precision. Power was set to 0.80 (Cohen, 1988) and a proportion of dishonest respondents of 0.20 was assumed (cf. Moshagen and Hilbig, 2016). On this basis, we calculated a sample size for a medium effect (*odds ratio* = 2.5; Rosenthal, 1996). A power analysis revealed a required sample size of at least 300 participants to detect a significant effect – given there is one (using the R Package RReg; Heck and Moshagen, in press). As a precaution we recruited 501 American participants from Amazon Mechanical Turk (*M*age = 33.92, *SD* = 10.5, range: 18–68; 213 females; all living in the United States; ethnicity: 339 Caucasians, 37 Asian Americans, 36 Asians, 31 Hispanics, 28 African Americans, 17 “Others,” 6 Indians, 5 without indication, and 2 Africans; 493 native English speakers; 430 employees). No participants were excluded.

### Procedure and Measures

Participants completed the study online, which took approximately 8 min. After participants agreed to take part in the study, we assessed their demographic measures (age, gender, home country, ethnicity, native language, and occupational status) and participants’ general and personal BJW, using the scales from Dalbert (1999). General BJW was measured with six items (α = 0.90; e.g., “I am convinced that in the long run, people will be compensated for injustices”) and personal BJW with seven items (α = 0.91; e.g., “I believe that, by and large, I deserve what happens to me”). Participants responded to all items on a 6-point Likert-type scale ranging from 1 (*strongly disagree*) to 6 (*strongly agree*). After 60 distractor items (PANAS-X, Watson and Clark, 1999), participants were introduced to a coin-toss game, where everyone who flipped the winning coin side entered a raffle and had a chance of winning a $100 voucher for Amazon.com. We applied a common one-shot cheating paradigm in which participants self-report the outcome of tossing a coin to detect the amount of dishonest reporting (Rosenbaum et al., 2014). Therefore they were told that they would participate in a tossing game and should make sure that they had a coin, which they would later need. Then they were instructed that they should flip the coin one time and indicate whether heads or tails was facing upward. Half of them were randomly informed that everyone who throws heads would participate in a raffle and could win said voucher. The other half were randomly informed that tails was the winning side of the coin. As soon as the participants had a coin and were ready to toss it, they continued the survey and were asked to toss the coin, as well as to indicate if they had tossed the winning coin side and would take part in the raffle (“Yes”) or not (“No”). This completely anonymous setup ensures that the true outcome is only known to the participants, and that thereby they have the opportunity to cheat by claiming to have tossed the target outcome, independent of their actual toss. At the same time, through such misreporting, they can increase their chances to win the voucher, which serves as the incentive to cheat. The proportion of dishonest individuals can later be inferred from the observed responses at the aggregate level, given the known probability distribution of chance (0.5) when tossing a coin (Moshagen et al., 2014). In the end, as religiousness and general BJW have been found to be correlated (e.g., Hafer and Sutton, 2016), we included religion as a control variable – that is, participants reported the importance of religion in their everyday life ranging from 1 (*not at all important*) to 9 (*very important*). To conclude, we assessed how interested they were in winning the voucher, ranging from 1 (*not at all interested*), to 7 (*very interested*), if they had recently participated in a similar study, and if they knew the purpose of the study.

## Results

Overall, 63.3% of the participants reported having flipped the winning outcome, which is significantly higher than the expected probability of chance (50%), *t*(500) = 6.16, *p* < 0.001, *d* = 0.275, *CI_95%_* = [59.1%; 67.5%]. In comparison, in a previous study using a similar coin-toss game with an online (MTurk) sample (Schindler and Pfattheicher, 2017), 76.7% of the participants reported having flipped the winning coin-side. Hence, dishonest behavior can be observed through such studies although the actual amount of dishonesty can vary. All in all, half of the participants should have flipped the winning outcome anyway, so that only among the other half who flipped the losing outcome some might at all consider dishonest behavior. Following the guidelines of Moshagen and Hilbig (2016), calculations showed that an estimated 13.3% in our sample actually deceives (0.633 – 0.50) and the estimated proportion of wins through cheating amounts to 21.01% [(0.633 – 0.50)/0.633]. Further, (estimated) 26.6% [(0.633 – 0.50)/(1 – 0.50)] of the participants in our sample are prepared to cheat if they do not actually win.

Results of the bivariate correlation analyses can be seen in **Table 1**. The results indicated a significant positive correlation between general and personal BJW (*M*_general_ = 3.77, *SD* = 1.06; *M*_personal_ = 4.26, *SD* = 0.95). Further, in line with our hypothesis, there was a significant positive correlation between general BJW and the probability of flipping the target outcome. There was also a significant correlation between general BJW and the importance of religion, but none between personal BJW and the probability of flipping the target outcome. We further conducted logistic regression analyses to test the effect of general BJW under control of personal BJW and the importance of religion, as well as the effect of personal BJW. Results can be seen in **Table 2**.

**Table 1:** Bivariate correlations of the different measures (*N* = 501).

	*1.*	*2.*	*3.*	*4.*
(1) Coin Toss	1			
(2) General BJW	0.09^∗^ (0.18^∗^)	1		
(3) Personal BJW	0.03 (0.05)	0.54^∗∗^	1	
(4) Importance of religion	0.01 (0.02)	0.21^∗∗^	-0.03	1

^∗^*p < 0.05, ^∗∗^*p* < 0.01. The correlations between the coin toss variable and the other three variables in parentheses were conducted using the R package RReg (Heck and Moshagen, in press), which takes random noise due to coin-toss paradigms into account. The other correlations are simple correlations without adjustment*.

**Table 2:** Logistic regression results of participants’ coin toss outcomes as a function of general and personal belief in a just world and self-reported importance of religion (*N* = 501).

		Parameter estimates				
Model	Predictor	*B*	*SE*	*Z*	*p*	Odds ratio
(1)	General BJW	0.19 (0.55)	0.09 (0.30)	4.01 (3.39)	0.045 (0.038)	1.21 (1.73)
(2)	Personal BJW	0.05 (0.16)	0.09 (0.30)	0.49 (0.30)	0.625 (0.559)	1.005 (1.18)
(3)	General BJW	0.22 (0.57)	0.11 (0.31)	3.86 (3.40)	0.049 (0.045)	1.24 (1.77)
	Personal BJW	-0.06 (-0.07)	0.11 (0.28)	0.28 (0.06)	0.595 (0.808)	0.94 (0.93)
(4)	General BJW	0.19 (0.55)	0.10 (0.30)	3.99 (3.37)	0.046 (0.039)	1.21 (1.73)
	Religion	-0.02 (0.01)	0.10 (0.22)	0.04 (0.004)	0.841 (0.951)	0.98 (1.01)
(5)	General BJW	0.23 (0.57)	0.12 (0.31)	3.88 (3.38)	0.049 (0.046)	1.26 (1.77)
	Personal BJW	-0.07 (-0.07)	0.11 (0.28)	0.33 (0.06)	0.566 (0.807)	0.94 (0.93)
	Religion	-0.03 (0.01)	0.10 (0.22)	0.09 (0.004)	0.767 (0.948)	0.97 (1.01)
(6)	General BJW	0.24 (0.83)	0.12 (0.36)	4.32 (5.42)	0.038 (0.013)	1.27 (2.29)
	Personal BJW	-0.03 (-0.36)	0.11 (0.28)	0.09 (1.68)	0.765 (0.220)	0.97 (0.70)
	General^∗^Personal	0.10 (0.29)	0.07 (0.14)	2.08 (4.46)	0.150 (0.056)	1.11 (1.34)
	Religion	-0.03 (0.03)	0.10 (0.23)	0.10 (0.02)	0.755 (0.900)	0.97 (1.03)

*General BJW, personal BJW and religion were z-standardized; Religion refers to participants’ self-reported importance of religion. The logistic regression analyses in parentheses were conducted using the R package RReg (Heck and Moshagen, in press), which conducts robust regressions adjusting the random noise due to coin-toss paradigms. The other estimates are the results of simple binary logistic regression analyses*.

Moshagen and Hilbig (2016) state that typical analyses of binary cheating paradigms ignore that the observed win-response is contaminated by honest respondents, leading to substantially underestimated effects. Thus, to adequately test our hypothesis, we additionally conducted bivariate correlation and logistic regression analyses using the R package RRreg (Heck and Moshagen, in press). For a better comparison of this adjustment, **Tables 1**, **2** include the results of the uncorrected as well as the corrected and more robust results. Both approaches found the same pattern of results and significances, only the size of the estimates differ.

As expected, general BJW significantly predicted the probability of flipping the winning coin side, with higher levels leading to a higher probability. Given our hypothesis, this finding remains robust in terms of significance level controlling for personal BJW, the importance of religion and an interaction between general and personal BJW. None of these variables was significantly linked to the probability of flipping the target outcome. In line with our hypothesis, the findings of this study revealed that higher levels of general BJW were linked to an increased probability of flipping the target outcome, and that participants with a stronger general BJW were more inclined to state a winning toss and to thereby enhance their chance of winning a voucher. In contrast to our aforementioned assumption, there was no significant effect of personal BJW on the probability of reporting the winning coin-side.

## Discussion

In the present work, we wanted to explore possible maladaptive side-effects of facets of BJW. Specifically, we aimed to examine the influence of people’s dispositional general and personal BJW on (dis)honest behavior. Previous research found general BJW to be associated with antisocial tendencies like vengefulness, harsh social attitudes, hostile attributions, and delinquent intentions (e.g., Bègue and Muller, 2006; Sutton and Winnard, 2007; Dalbert, 2009; Hafer and Sutton, 2016). Accordingly, we hypothesized higher levels of general BJW to be linked to more dishonest behavior. Our study supported this idea: General BJW significantly predicted the probability of tossing the target outcome. This speaks for our idea that actual dishonest behavior is higher when people have a strong belief that the world is a just place in general. In sum, our study supports the novel role of general BJW as a relevant predictor for dishonest behavior and that it can lead to maladaptive side-effects. Moreover, this effect was found to be independent from personal BJW, the interaction between personal and general BJW and the importance of religion. Although previous research led us to the assumption that personal BJW and dishonesty would be negatively correlated, our study could not support this assumption.

All in all, our results were able to show a maladaptive side-effect of BJW. Even though BJW normally serves an adaptive function, at least the general facet of it can have maladaptive consequences. This is in line with the aforementioned research concerning meaning making, because meaning making (which can be used to restore a BJW after an experienced unjust or traumatic event) actually serves an adaptive function but can also present harmful side-effects (e.g., Kernan and Lepore, 2009). In regards to meaning making, it seems logical that the adaptive function mostly concerns a personal BJW (and not a general facet) because it is especially doable and helpful to sustain a belief in a personally, instead of a generally, just world. The same could be true for the belief in a just world. Further, this supports the aforementioned assumptions that people with stronger levels of general BJW do not value the contract between them and the world and that the moral costs for cheating seem to be lower for them. It also fits our thought that perhaps general BJW is not as socially desirable as personal BJW, and that therefore people who still indicate high scores do not feel the need to convey a desirable outcome – and therefore do not feel afraid to cheat.

Interestingly, the incentive for participating in a raffle for a voucher implies not only a possible gain for oneself, but also a reduced winning likelihood for the other participants. That is, the more people cheated to win the voucher, the lower everyone’s probability of winning – although it is not clear to what extent people even have this in mind while cheating.

From a theoretical perspective, there are some possible explanations for the found effects. We think that the link between general BJW and dishonest behavior can be best explained in terms of justifications (cf. Shalvi et al., 2011). As mentioned before, people confronted with a chance to cheat have to decide whether the potential gain (in our case, the possibility of winning a voucher) outweighs the (moral) cost of cheating (e.g., an impaired positive self-concept; e.g., Mazar et al., 2008). Therefore, people need justifications for cheating so that they can cheat (and gain) without having to suffer moral costs (e.g., Shalvi et al., 2011). People with a high general BJW are assumed to perceive the world as generally just. This justice in the world can be seen as independent of their own behavior; that is, being dishonest does not affect what other people get, because others are believed to get what they deserve anyway, so that they either deserve (in this case) winning the voucher or not, independent of others’ dishonest behavior. This in turn reduces moral costs of cheating, because in such circumstances cheating is not believed to directly cause harm. Just as well, people with a high general BJW could believe that even if their cheating harmed others, they would be compensated elsewhere so that things would even out for those people in other ways. According to this reasoning, perceiving the world as a just place in general might serve as justification for dishonesty or lower costs of dishonesty. Therefore, people would be able to cheat without impairing their positive self-concepts. Due to that, the gain of cheating (possibly winning a voucher) is the same for everyone, but people with stronger levels of general BJW have lower costs than people with lower levels or people with stronger levels of personal BJW. Pondering the gain and the cost of cheating, people with strong general BJW probably often decide to cheat because the gain outweighs the costs.

Another explanation could be that people with a strong general BJW perceive the harm caused by their cheating as deserved, because others could have cheated as well; therefore, their reduced odds of winning are their own faults if they do not cheat themselves. In line with this, a strong general BJW (which correlates with victim blaming, e.g., Dalbert, 2009) could even lead to (proactively) blaming victims of one’s own dishonest behavior, because in a generally just world only blameworthy or unsympathetic people should actually fall prey to others’ dishonesty and nothing undeserved would happen. Unfortunately, our study is not able to distinguish between these different possibilities. Future research should therefore try to explore the underlying reasons for the present effect, for example, by manipulating the consequences of own dishonesty, the existence of justifications, perceived feelings of deservingness, or by assessing participants’ reasoning.

Previous research from Sutton and Winnard (2007) found the aforementioned positive relation between BJW for others and delinquent intentions only controlling for personal BJW, thus there was a suppressor effect. However, our study did not find such a suppressor effect but rather a positive zero-order correlation between general BJW and dishonesty independent of personal BJW. It is possible that this suppressor effect is unique for BJW for others (and not for general BJW) because of the scale’s slightly different instructions (concerning the inclusion or exclusion of oneself in a generally just world) and items. Still, due to the relatedness of BJW for others and general BJW, it can be assumed that prior findings concerning BJW for others should apply for general BJW as well, and findings concerning general BJW should apply for BJW for others. Therefore, it would be interesting if future research could find the same positive correlation measuring not general BJW but BJW for others (or even implicit measurements) as well as using varying settings and methods (for instance using varying incentives to cheat).

Previous findings further suggested a negative link between personal BJW and dishonesty, because high personal BJW was assumed to be linked to striving for justice, trust, and prosocial behavior. However, our findings could not support this assumption. Thus, given that there are good theoretical reasons for assuming personal BJW as a negative predictor for dishonesty, the present findings should instead be interpreted with caution. Maybe our dependant variable is not as valid as we thought. On the other hand, it could also be that participants did not immediately recognize that their cheating behavior even had an impact on other people, and without such negative consequences for others, they did not recognize their behavior as unjust. It is also possible that a single chance of winning a voucher is not strong enough to trigger striving for justice, so that personal BJW may only have effects on more important or more consequential matters. We should therefore have explicitly assessed whether participants really recognized the consequences of their (dis)honest behavior and how high they rated their chance of winning. In retrospect, more control variables would have been helpful. Although participants indicated that they were strongly interested in winning the voucher (*M* = 6.41, *SD* = 1.18), their dishonesty did not fully determine their gain; it only slightly increased their winning chances. Maybe personal BJW would have had an influence in a setting were people’s (dis)honesty actually specified their outcome (or others). For instance, the aforementioned study form Bègue (2014) could show that BJW for the self increased prosocial behavior, but concerning money that could directly be donated to an organization. Maybe personal BJW only has implications on behavior in situations which have directly measurable or observable impacts on oneself or other people. All in all, it would be important to replicate the present findings with different operationalizations of actual dishonest behavior, different samples (especially regarding online versus laboratory studies), and more control variables.

Although we theoretically propose a causal effect of general BJW on dishonesty, the present finding is only correlative in nature, which is clearly a limitation. After all, even the opposite direction – participants’ dishonesty influencing their self-reported general BJW- could be possible. Even though we assessed participants’ beliefs before they were introduced to the coin-toss paradigm, we cannot with any reasonable certainty assume a causal effect. It may even be plausible that participants who in the beginning described their general BJW as very strong later only cheated because their self-reported belief let them feel entitled to do so. Therefore, an experimental manipulation of participants’ BJW would be more than appropriate.

Still, even if there are some limitations to our study, our findings are very interesting and we think that the present work is certainly stimulating for future research. All in all, we were able to show that general BJW can have maladaptive side-effects and not only influence people’s attitudes and tendencies but even their actual everyday behavior. This is exciting on one hand because there is a vast difference between attitudes and actual actions, and on the other because BJW normally primarily serves an adaptive function. Future research could try to replicate our results, especially while optimizing the aforementioned problems and with a stronger focus on personal BJW.

## Ethics Statement

The study was conducted in full accordance with the Ethical Guidelines of the German Association of Psychologists (DGPs) and the American Psychological Association (APA). Moreover, by the time the data were acquired it was also not customary at Kassel University, nor at most other German universities, to seek ethics approval for simple studies on personality and attitudes. Therefore, ethical approval was not required for this study in accordance with the national and institutional guidelines. The study exclusively makes use of anonymous questionnaires. No identifying information was obtained from participants. Every participant had to read (and agree to) two questions concerning their consent. They were thereby explicitly informed that all data are treated confidentially and that they may withdraw from the study at any time without giving explanation. Moreover, it was possible for participants to easily withdraw from the study at any time simply by closing the internet browser.

## Author Contributions

All authors fulfill the mentioned authorship criteria. Therefore, all authors contributed to all parts of the work. Hence, all authors were involved with the conception, acquisition, analysis, interpretation, drafting and revising of the work. Concluding, all authors gave their final approval of the version to be published and agree to be accountable for all aspects of the work.

## Conflict of Interest Statement

The authors declare that the research was conducted in the absence of any commercial or financial relationships that could be construed as a potential conflict of interest.
